# The synthetic cannabinoids menace: a review of health risks and toxicity

**DOI:** 10.1186/s40001-023-01443-6

**Published:** 2024-01-12

**Authors:** Ayman Alzu’bi, Fatimah Almahasneh, Ramada Khasawneh, Ejlal Abu-El-Rub, Worood Bani Baker, Raed M. Al-Zoubi

**Affiliations:** 1 Department of Basic Medical Sciences, Faculty of Medicine, Yarmouk University, Irbid, 211-63 Jordan; 2 Surgical Research Section, Department of Surgery, Hamad Medical Corporation & Men‘S Health, Doha, Qatar; 3 Department of Biomedical Sciences, QU-Health, College of Health Sciences, Qatar University, Doha, 2713 Qatar; 4 Department of Chemistry, Jordan University of Science and Technology, P.O.Box 3030, Irbid, 22110 Jordan

**Keywords:** Synthetic cannabinoids, Spices, K2, Designer drugs, Toxicity

## Abstract

Synthetic cannabinoids (SCs) are chemically classified as psychoactive substances that target the endocannabinoid system in many body organs. SCs can initiate pathophysiological changes in many tissues which can be severe enough to damage the normal functionality of our body systems. The majority of SCs-related side effects are mediated by activating Cannabinoid Receptor 1 (CB1R) and Cannabinoid Receptor 2 (CB2R). The activation of these receptors can enkindle many downstream signalling pathways, including oxidative stress, inflammation, and apoptosis that ultimately can produce deleterious changes in many organs. Besides activating the cannabinoid receptors, SCs can act on non-cannabinoid targets, such as the orphan G protein receptors GPR55 and GPR18, the Peroxisome Proliferator-activated Receptors (PPARs), and the Transient receptor potential vanilloid 1 (TRPV1), which are broadly expressed in the brain and the heart and their activation mediates many pharmacological effects of SCs. In this review, we shed light on the multisystem complications found in SCs abusers, particularly discussing their neurologic, cardiovascular, renal, and hepatic effects, as well as highlighting the mechanisms that intermediate SCs-related pharmacological and toxicological consequences to provide comprehensive understanding of their short and long-term systemic effects.

**Graphical Abstract:**

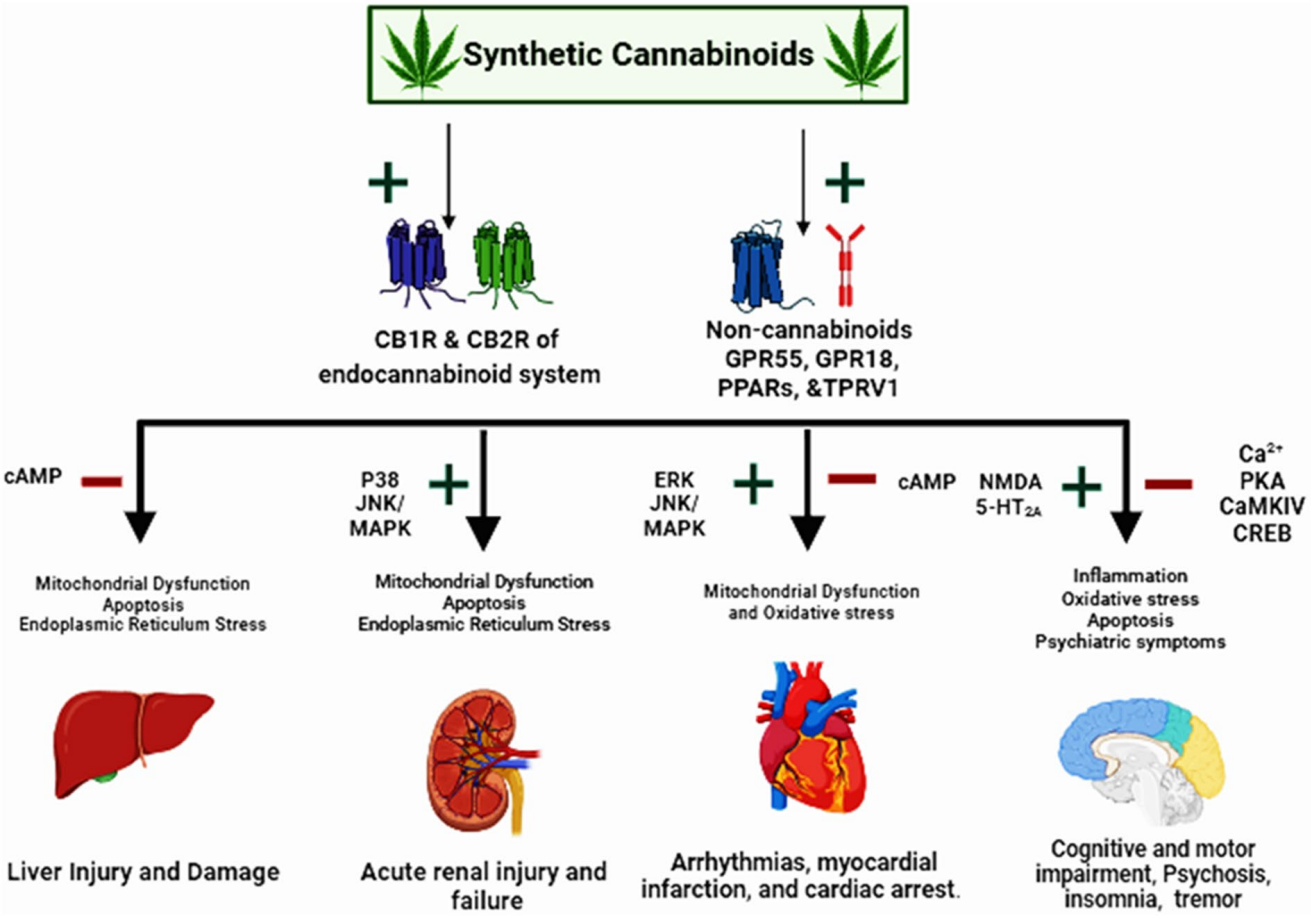

## Introduction

The recreational use of synthetic cannabinoids (SCs) has markedly increased in recent years. Numerous reports have linked SCs consumption to the incidence of various adverse health effects, turning their widespread use into major public health concern. These compounds are chemically designed to mimic the effects of Δ9-tetrahydrocannabinol (THC), the main psychoactive ingredient of marijuana [1, 2]. However, most SCs exhibit much higher binding affinities to the cannabinoid receptors 1 and 2 (CB1R and CB2R) when compared to THC [3, 4]. In addition, beyond binding the CB1R and CB2R, it has been demonstrated that SCs also interact with non-cannabinoid targets [5, 6] which may result in distinct pharmacologic effects as well as diverse toxicity profile.

**Fig. 1:**
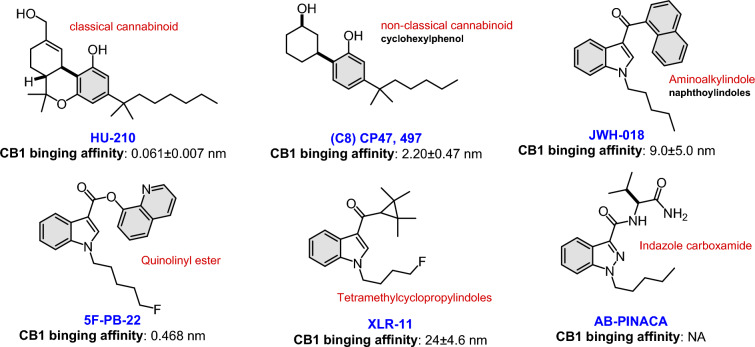
Structure and binding affinity of popular synthetic cannabinoids [5, 9]

SCs are found in illicit drug market in products available with several brand names, such as Spice, K2, Black Mamba, fake weed, and joker. The composition of these products usually has unpredictable nature, but they are commonly mixtures of several potent synthetic CB1R agonists, such as AB‑CHMINACA, AB‑FUBINACA, AB‑PINACA, AMP-FUBINACA, 5-FLOUROMDMB PICA, 5-FLOUROMDMB-BUTINACA, AM‑2201, CP-47, CP-497 HU210, JWH‑018, JWH‑073, JWH‑200, UR‑144, FUB-144, XLR‑11 as well as many other names (Fig. 1). Although many SCs are placed under the schedule I drugs category by the US Drug Enforcement Administration, identified as unsuitable for medical use as they possess a high potential for abuse and addiction, these only represent a few among several hundreds of largely unknown and newly created compounds estimated by the United Nations Office to be circulating as drugs of abuse worldwide [7, 8].

Over the past decade, numerous reports have linked SCs ingestion with the emergence of a wide range of serious adverse health effects. These effects are not only limited to the central nervous system (CNS), but also recognised in other body targets, including cardiovascular, renal, respiratory, digestive and immune systems. The most commonly reported toxic effects linked to SCs use include agitation, anxiety, drowsiness, nausea, vomiting, depressed breathing, tachycardia, hypertension, muscle twitches, as well as more dangerous effects, such as psychosis, cognitive impairment, stroke, seizures, cardiac complications, acute renal failure, and acute hepatic injury [1, 10, 11]. Furthermore, reports of overdose deaths following SCs ingestion have markedly increased in recent years [12–15].

Similar to THC, SCs exert their actions mostly by binding to CB1R and CB2R. However, SCs act as full agonists of these receptors and typically exhibit much higher potency and efficacy than the partial agonists THC [3, 4]. CB1R are primarily expressed in the CNS; however, they are also detected at lower but functionally significant levels in most peripheral tissues, including heart, liver, lung, kidney, vascular endothelium, and reproductive system [16, 17]. On the other hand, CB2R are mainly expressed in immune and immune-derived cells, but their presence has been also established in the brain and various peripheral tissues [18, 19]. Activation of CB1R and CB2R inhibits adenylyl cyclase leading to the consequent modulation of ion-channel function [20]. In addition, on the basis of the cell and ligand types, it has appeared that the signaling of CB1R (and to lesser degree CB2R) is also implicated in the modulation of multiple intracellular pathways, including mitogen-activated protein kinase (MAPK) pathway, phosphoinositide 3-kinase (PI3K)/protein kinase B (Akt) pathway, ceramide signaling, and many others [21–23]. Therefore, the wide distribution of cannabinoid receptors and the variety of associated signaling pathways have been described as the main biological elements for various SCs-related toxicities reported in literature [24, 25]. Furthermore, beyond binding CB1R and CB2R, it has been proposed that SCs pharmacological actions may also be mediated by several cannabinoid-independent receptors, such as the transient receptor potential vanilloid 1(TRPV1), the peroxisome proliferator-activated receptor (PPARs) family, and the orphan G protein-coupled receptors 55 (GPR55), 18 (GPR18) and 119 (GPR119) [26–30]. These receptors are abundantly expressed in the CNS and peripheral tissues, and have been shown to modulate multiple intracellular signaling pathways independently of CB1R and CB2R, which may reasonably explain the diverse toxicity profile of SCs [6, 24].

This review will shed light on recent updates of adverse health events that are linked to the consumption of SCs and explain the possible mechanisms of SCs-related intoxications as described in the literature. This can improve SCs risk awareness and assist health care decision makers in finding proper treatment plans for addicted patients to improve their quality of life and reduce the mortality rate related to SCs abuse. We searched the PubMed MEDLINE database of the US National Library of Medicine. The MeSH (medical subject headings) terms used in the search strategy were synthetic cannabinoids-related toxicities, synthetic cannabinoids and neurotoxicity, synthetic cannabinoids and renal toxicity, Keywords used were cannabinoid receptors, synthetic cannabinoids, K2, spice. We included the most recent preclinical and clinical studies related to the review topic, and only English articles were selected.

### Neurologic and psychiatric effects

The neurologic and psychiatric toxic effects of SCs are multiple and well-documented in the literature. The nervous system is significantly affected by SCs, that is mainly due to the extensive distribution of cannabinoid receptors in several brain areas, such as the cerebral cortex, hippocampus, basal ganglia, amygdala, and cerebellum [26]. SCs use has been associated with several neurological perturbations, including drowsiness, dizziness, lethargy, confusion, anxiety, delirium, seizures and convulsions, and impaired motor performance [32]. Chronic use of SCs has been particularly associated with cognitive effects, including impairment of attention, learning and memory, mental flexibility, and emotional processing [25, 33]. In addition, it has been demonstrated that SCs use is correlated with an increased risk of psychiatric disorders [34]. Psychosis, in particular, has been described as the most serious toxic effect of long term use of SCs that is not reported with natural cannabinoid use; this may be due to the protective effect of cannabidiol, which is a component of the natural compounds, but is not found in psychoactive SCs products [35].

Activation of CB1R and CB2R in the presynaptic membrane stimulates pertussis toxin-sensitive G proteins (*G*_*i/o*_), which inhibits adenylyl cyclase and leads to a decrease in protein kinase A activity. Activation of CB1R, through the βγ subunits, also triggers the inhibition of voltage-gated Ca^2+^ channels with simultaneous activation of inwardly rectifying K^+^ currents. These signaling components inhibit the neurotransmitters release into the synaptic cleft, thus influencing the excitatory and inhibitory synaptic transmission [36]. At the postsynaptic membrane, activation of CB1R is responsible for the activation of MAPK pathway, which can further trigger the activation of extracellular signal regulated kinases 1 and 2 (ERK1/2) as well as regulate certain nuclear transcription factors which can influence the gene expression profile [37].

Some of the underlying mechanisms of SCs-induced neurotoxicity have been evidenced in literature. For example, in the study by Basavarajappa and colleagues, the deleterious effects of JWH-081 on murine hippocampal function involved CB1R-mediated impairment in CaMKIV and CREB phosphorylation [31]. Tomiyama and Funada found that SCs induce apoptosis of mouse forebrain primary neurons through a CB1R-mediated caspase-3-dependent mechanism [38]. Other studies investigating the mechanisms of SCs neurotoxicity reported reduced mitochondrial membrane potential in neuroblastoma cell lines [39]. In addition, impaired mitochondrial activity and mobility were identified as mechanisms of SCs toxicity. These processes involve intra-mitochondrial Gαi protein activation and consequent inhibition of soluble-adenylyl cyclase (sAC), which inhibits protein kinase A (PKA)-dependent phosphorylation in the mitochondrial electron transport system, resulting in impaired cellular respiration [40, 41]. Exposure of neuroblastoma SH-SY5Y cells to APINACA increased the formation of reactive oxygen species (ROS) and the expression of CB1R, MAPK8, IL-6 and TNF-α [42]. Similarly, JWH-018 induced oxidative stress in SH-SY5Y cells [43]. Coccini and colleagues investigated the effects of MAM-2201 on cell viability, mitochondrial function, apoptosis and morphology of cells of the nervous system and found these effects to be mediated by CB1R in astrocytes and unrelated to CB1R in neurons [44]. The mechanism of SC-induced seizures is not well-defined, but strong binding of the CB1R receptors seems to be involved [45].

The molecular mechanisms underlying the pro-psychotic effects of SCs have been proposed as a result of the action of these substances at CB1R, which can modulate the functions of neurotransmitter systems known to be associated with the pathophysiology of schizophrenia and psychosis [46]. SCs stimulate the dopamine (DA) release and increase the firing activity of DA-expressing neurons. For example, the SCs JWH-018, AKB48, and 5F-AKB48 were found to elevate the DA level in nucleus accumbens [39, 47]. Administration of the SCs WIN55212-2 and CP55940 in rats increased the firing rate and bursting activity of A10 DA neurons [48]. Other neurotransmitters involved in the pathophysiology of schizophrenia, such as serotonin and glutamate, have been also associated with the psychotic effects of SCs. SCs upregulate 5-HT_2A_ receptors through activation of CB1R and ERK1/2 signaling pathway and increase the interaction of 5-HT_2A_ receptors and dopamine receptors in the prefrontal cortex [49, 50]. CB1R agonists were found to alter the function of NMDA receptors, which are known to be associated with psychiatric disorders. This effect is mediated by attenuation of glutamate release [51] and events downstream from CB1R signaling [52].

As mentioned above, beyond binding CB1R and CB2R, SCs can also mediate their action by targeting a number of non-cannabinoid receptors. Among these, the orphan G protein receptors GPR55 and GPR18, the nuclear hormone receptors PPARs, and the ion channel TRPV1 are broadly expressed in the CNS and are involved in the modulation of multiple intracellular signaling pathways. The GPR55 is coupled with G12/13 proteins, being able to increase the intracellular calcium levels via the activation of RhoGTPase nucleotide exchange factors [26, 53]. The activation of GPR55-Rho signalling pathway can disturb the redox balancing in the brain, trigger neuroinflammation, and damage the blood brain barrier integrity [54–56]. In contrary, activation of GPR55 can also trigger protective mechanisms in the brain that can balance and curb the high level of oxidative stress and inflammation depending on the downstream players, such as Nuclear factor erythroid 2-related factor 2 (NRF2) and ERK [57]. The GPR18 interacts preferably with CB2R causing the activation of microglia and triggering neuroinflammation [58]. SCs may also activate Peroxisome Proliferator-activated Receptors (PPARs), a family of nuclear hormone receptors, to form heterodimers with the retinoid X receptor and stimulate gene expression by binding to specific DNA sequences known as PPAR response elements [59]. SCs found to activate the three different isoforms of PPARs; PPARα, PPARβ, and PPARγ, which induce the transcription of key factors involved in regulating inflammation, metabolism, and oxidative stress [59]. The activation of PPARs by SCs instigates many neuroprotective mechanisms by reducing the level of master inflammatory cytokines; NF-Kb and Nrf2/CREB, and scavenging excess level of free radicals. Interestingly, there is a molecular connection between CB1R and PPARs that can dictate the ultimate effects of SCs, intense activation of CB1R by SCs can disturb the neuroprotective effects exerted by PPARs and incite intense inflammatory and oxidative stress responses [60]. Although SCs exhibit limited efficacy in opening TRPV1 channels, some SCs such as XLR-11 were shown to induce a significant activation of these channels and promote neuronal uptake of Ca2 + [61].

### Cardiovascular effects

The association between SCs use and the incidence of cardiovascular complications has been corroborated thoroughly by many studies. Numerous case reports and clinical studies have linked SCs ingestion with serious acute cardiac toxicities, including hypertension, tachycardia, arrhythmias, myocardial infarction, and cardiac arrest. Although hypertension and tachycardia are the most commonly reported cardiac complications related to SCs ingestion [11, 62–66], more serious cardiovascular events have been also described in literature. Current epidemiological data suggest that acute and chronic use of SCs has been linked to various arrhythmias that include sinus bradycardia, second-degree atrioventricular block, ventricular fibrillation, and atrial fibrillation [42]. Mir et al., reported three cases of adolescent patients, who complained of chest pain only 1 day after smoking K2 and were diagnosed with acute myocardial infarction based on electrocardiogram changes (ECG) and elevated troponin levels [66]. Subsequent cases of acute myocardial infarction and cardiac arrest have been also reported after K2 abuse in healthy young teenagers or young adults [67–70]. In addition, several cardiovascular fatalities associated with SCs abuse have been also reported in literature [15, 71–75].

The mechanisms of SCs-induced cardiotoxicity have been suggested to involve activation of CB1R, which are extensively expressed in the cardiovascular system [76]. In this regard, several studies revealed that the activation of CB1R mediates a cascade of events that eventually lead to myocardial injury and disturb the cardiovascular system dynamics [77]. It has been demonstrated that the interaction of CB1R receptors with their ligands is associated with the activation of p38 kinase and the mitogen-activated protein kinase (MAPKs) JNK, followed by subsequent induction of apoptosis signaling pathway. Furthermore, the disturbance in Redox signaling increased the level of reactive oxygen species that initiate an oxidative tissue stress and damage in the cardiac tissues. The possible role of activating ERK kinases 1 and 2 in inducing cardiac hypertrophy has been also suggested [76]. CB1R receptors are predominantly localized within the mitochondria, where their activation may alter the mitochondrial biogenesis and Redox oxidative balance. The exogenous activation of myocardial CB1R receptors is possibly implicated in changing mitochondrial dynamics by disturbing the mitochondrial respiratory chain complexes, inhibiting the synthesis of essential mitochondrial enzymes, and the subsequent loss of inner mitochondrial membrane potential, which initiates mitochondrial oxidative stress and cellular apoptosis [78]. In congruence with that, Alexandre and co-authors have observed a remarkable increase in mitochondrial membrane potential by 1 pM and 1 μM using THJ-2201 and 5F-PB22, respectively, suggesting a profound impairment of mitochondrial activity [79]. These studied SCs induced a transient mitochondrial membrane hyperpolarization and increased intracellular ATP levels, which subsequently ensued massive chromatin condensation and caspase-3 activation that triggered the activation of cellular apoptosis [80]. The high intracellular ATP levels induced by SCs could be possibly attributed to the inhibition of adenylate cyclase activity [81]. CBR1 receptor activation is also known to inhibit adenylate cyclase activity that will be associated with downregulation of cyclic adenosine monophosphate (cAMP) production, accumulation of ATP molecules, and decreased ATP consumption rate [82]. Taken together, these data suggest that SCs consumption causes substantial impairment of mitochondrial dynamics and enkindles destructive oxidative stress that leads to myocardial damage and serious complications that can be critical and carried a high mortality risk. On the other hand, the cardiovascular complications among SCs consumers can be also a direct consequences of intense activation of sympathetic nervous system and inhibition of the parasympathetic nervous system as a result of CB1R receptors activation in the brain and locally within the heart [83]. Triggering powerful sympathetic stimulation can negatively affect the heart contractile cells due to unbridled increase in the cardiac contractility, workload, and oxygen demands. The consequences of strong sympathetic activity can be dangerous, leading to various remodeling changes that can initiate serious cardiac events.

In the similar fashion to CNS, non-cannabinoids targets can mediate many pharmacological effects of SCs in the cardiovascular system. GPR55 and GPR18 are widely expressed in the cardiovascular system, and their activation by SCs can excite salutary or harmful events depending on the downstream targets [84]. It has been shown that the activation of GPR55 in the heart can initiate vaso-relaxatioin effect, slow-down the development of remodeling changes in the heart, such as hypertrophy, and downregulate the levels of extracellular matrix factors and inflammatory cytokines [85]. These mechanisms suggested that the long-term pernicious effects of SCs on the heart depend on the failure in creating a balance between cannabinoids and non-cannabinoids signaling pathways of SCs [23]. Furthermore, the deleterious effects of SCs on heart can be more intense when higher doses, because these protective non-cannabinoids signaling pathways can be paralyzed with higher doses of SCs and longer duration of exposure, while the harmful cannabinoid signaling pathway, particularly CB1R-mediated signaling, became more active and prominent [76].

### Renal effects

Although renal toxicities are considered uncommon complications associated with SCs usage, accumulating evidence suggests that cannabis or its synthetic analogues may have deleterious effects not only on kidney function in patients with pre-existing kidney disease, but also on healthy kidneys [86]. In particular, a significant number of case reports anticipated possible association between acute kidney injury (AKI) and SCs ingestion in healthy adolescents and adults who do not have a previous medical history of kidney diseases [87–91]. Those SCs consumers usually presented to the emergency department complaining of new onset of intense nausea, vomiting, and abdominal or flank pain, with elevated serum levels of creatinine and urea. The clinical records of some cases demonstrated the existence of acute tubular necrosis and acute interstitial nephritis detected upon histological examination of their renal biopsies [11].

The exact pathophysiology of SCs-associated AKI remains largely unknown. However, functional CB1R and CB2R were detected in various renal cells, including cells of glomeruli, proximal tubules, distal tubules, the loop of Henle, and collecting ducts [92–94], with substantial concentrations of endocannabinoids AEA and 2-AG also found in renal tissue [95]. In addition, several pharmacological studies have shown that the ECs play an essential role in regulating renal homeostatic processes, particularly urinary protein excretion, tubular sodium transport glomerular filtration, and renal vascular hemodynamics through the activation of CB1R receptors. Therefore, given the role of renal ECs under physiological conditions [86] as well as its role in the pathogenesis of several kidney diseases [96], it is reasonable to expect that dysregulation of renal ECs by exogenous pharmacological agents, such as SCs, can possibly lead to several pathophysiologic consequences. In this respect, Silva et al. demonstrated in a series of in vitro studies that SCs, such as XLR-11, AB-FUBINACA, JWH-122, and THJ-2201, through activation of CB1R and CB2R at in vivo relevant level, lead to disruption of mitochondrial function in human proximal tubule (HK-2) cells, which involves a transient hyperpolarization of the inner mitochondrial membrane and accumulation of intracellular ATP, with consequent triggering of renal cells apoptosis [80, 97]. The activation of CB1R and CB2R as initial step to start consequent events that ultimately lead to kidney injury and dysfunction has been also described in various preclinical disease models. For example, Lim et al. revealed that the activation of renal CB1R receptors can induce apoptosis in human proximal tubule cells mediated by activating the endoplasmic reticulum (ER) stress signaling pathway [98]. In addition, the ECs through CB1R, was found to promote cisplatin-induced kidney injury, mainly by augmenting p38 and JNK MAPK activation and enhancing the interrelated inflammatory and oxidative stress responses [99]. Finally, it has been also suggested that SCs ingestion can induces harmful renal effects indirectly and independently of CB1R and CB2R activation. SCs can be subjected to biotransformation and functional modifications in the liver leading to the production of many circulating toxic compounds that have the potential to cause nephrotoxicity by various stress pathways [100].

### Hepatic effects

The possibility that SCs use may cause liver damage has been described in human and animal models [12, 101, 102]. Numerous case reports have demonstrated an association between liver injury and SCs ingestion. Many patients with history of chronic SCs use were admitted to the hospital emergency care units suffering from toxic hepatitis with symptoms, such as abdominal pain, vomiting, and fatigue. Their laboratory findings revealed elevated liver enzymes, such as aspartate aminotransferase, alanine aminotransferase, alkaline phosphatase, and bilirubin, which can indicate the presence of serious liver injury [103–106]. In addition, liver damage and failure have been listed as the main cause of death in postmortem case reports with known history of SCs ingestion [107].

The exact mechanisms of hepatotoxicity and liver damage due to SCs use are not completely known. Many studies linked SCs-related hepatotoxicity with oxidative stress damage of mitochondria and ER [103]. As an evidence of oxidative stress involvement, it has been found that prompt treatment with N-acetylcysteine (hepatoprotective antioxidant agent) can significantly restore the oxidative capacity of the liver and improve the clinical outcomes [106]. Along with oxidative stress, intensive inflammatory response was shown as a key mediator in SCs-induced hepatotoxicity in vivo [102]. Although hepatotoxicity is believed to be primarily mediated by secondary cytotoxic effects of SCs and their metabolites, direct activation of CB1R and CB2R and dysregulation of ECs in the liver are also theorized as potential mechanism. CB1R and CB2R are expressed in various cell types of liver [108, 109], and were described to be involved in the pathogenesis of many chronic liver diseases [110]. In addition, inhibition of CB1R was found to play an essential role in the reduction of interrelated inflammatory response in toxin-induced liver injury [111]. Therefore, we can probably assume that SCs activation of CB1R and CB2R and their downstream signaling may also contribute to the emergence of liver toxicity and subsequent liver damage and failure. Besides activating CB1R and CB2R in hepatocytes, SCs can activate and modulate the expression of PPARs, particularly PPARα, which can modulate the activity of enzymes responsible of driving fatty acid oxidation and ketone bodies production in the liver, thus increasing the risk of developing metabolic acidosis [60]. On contrary, PPARα can enhance the anti-inflammatory and antioxidant signalling in the liver, which was also found to be demoted by the activation of CB1R by SCs [112].

### Effects on other organs

In addition to the abovementioned, the binding of SCs to CB1R receptors can negatively impact the pulmonary functions. SCs can increase the incidence of alveolar damage or haemorrhage and acute respiratory failure mediated by CB1R-induced inflammation and immune cells infiltrates [113]. On the other hand, CB2R can reduce the risk of developing acute lung injury due to bacterial infection [114]. Although the immunomodulatory effects of SCs are not fully understood, it has been suggested that activating CB1R and CB2R by SCs can reprogram the immune system by polarizing cytokines secretion causing the inhibition of T-cells, B-cells, natural killers, monocytes, and granulocytes [115]. Interestingly, SCs have been reported to produce promising effects for treating ocular conditions, such as glaucoma and ocular surface injury, as they can lower ocular hypertension and ocular inflammation [116]. However, their use is not recommended due to high risk of serious systemic and ocular side effects. SCs can cause ocular motility deficits, neuro-retinal dysfunction, and impaired visual acuity [117].

## Conclusion

Synthetic cannabinoids (SCs) recreational use is an illegal consumption that spreads rampantly worldwide, and the most targeted age groups are teenagers. Despite the huge efforts to crackdown on SCs consumption, the rate of recreational use is increasing as many cheap brands are handy and easily accessible. SCs abuse triggers multisystem intoxication that can be severe and lead to death, SCs-related toxicities were mediated by activating cannabinoids (CB1R and CB2R) and non-cannabinoids targets (such as GPR55) that increased the levels of ROS and inflammatory cytokines and disturbed the anti-inflammatory and antioxidant mechanisms. The imbalance between cannabinoids and non-cannabinoids mediated signalling of SCs seems to determine the severity of SCs-related toxicities. Keeping updates about pathological implications of SCs abuse can help in revamping the existing health care services and the associated addiction and harm reduction interventions.

## Data Availability

The data that supports the findings in this study are available from the corresponding authors upon reasonable request.

## References

[1] Castaneto MS, Gorelick DA, Desrosiers NA, Hartman RL, Pirard S, Huestis MA (2014). Synthetic cannabinoids: epidemiology, pharmacodynamics, and clinical implications. Drug Alcohol Depend.

[2] Fattore L, Fratta W (2011). Beyond THC: the new generation of cannabinoid designer drugs. Front Behav Neurosci.

[3] Vardakou I, Pistos C, Spiliopoulou Ch (2010). Spice drugs as a new trend: Mode of action, identification and legislation. Toxicol Lett.

[4] Auwärter V, Dresen S, Weinmann W, Müller M, Pütz M, Ferreirós N (2009). ‘Spice’ and other herbal blends: harmless incense or cannabinoid designer drugs?. J Mass Spectrom.

[5] Hess C, Schoeder CT, Pillaiyar T, Madea B, Müller CE (2016). Pharmacological evaluation of synthetic cannabinoids identified as constituents of spice. Forensic Toxicol.

[6] De Petrocellis L, Di Marzo V (2010). Non-CB1, Non-CB2 receptors for endocannabinoids, plant cannabinoids, and synthetic cannabimimetics: focus on G-protein-coupled receptors and transient receptor potential channels. J Neuroimmune Pharmacol.

[7] United Nations Office on Drugs and Crime. Synthetic cannabinoids: key facts about the largest and most dynamic group of NPS. UNODC https://www.unodc.org/documents/scientific/Global_SMART_Update_13_web.pdf.2015

[8] US Drug Enforcement Administration. Drug scheduling. https://www.dea.gov/drug-scheduling. Accessed 19 Nov 2018

[9] Banister SD, Moir M, Stuart J, Kevin RC, Wood KE, Longworth M (2015). Pharmacology of Indole and Indazole Synthetic Cannabinoid Designer Drugs AB-FUBINACA, ADB-FUBINACA, AB-PINACA, ADB-PINACA, 5F-AB-PINACA, 5F-ADB-PINACA, ADBICA, and 5F-ADBICA. ACS Chem Neurosci.

[10] Alves VL, Gonçalves JL, Aguiar J, Teixeira HM, Câmara JS (2020). The synthetic cannabinoids phenomenon: from structure to toxicological properties. A review. Criti Rev Toxicol.

[11] Alipour A, Patel PB, Shabbir Z, Gabrielson S (2019). Review of the many faces of synthetic cannabinoid toxicities. The Mental Health Clinician.

[12] Armstrong F, McCurdy MT, Heavner MS (2019). Synthetic cannabinoid-associated multiple organ failure: case series and literature review. Pharmacotherapy J Human Pharmacol Drug Therapy..

[13] Kasper AM, Ridpath AD, Gerona RR, Cox R, Galli R, Kyle PB (2019). Severe illness associated with reported use of synthetic cannabinoids: a public health investigation (Mississippi, 2015). Clin Toxicol.

[14] Shanks KG, Clark W, Behonick G (2016). Death associated with the use of the synthetic cannabinoid ADB-FUBINACA. J Anal Toxicol.

[15] Adamowicz P (2016). Fatal intoxication with synthetic cannabinoid MDMB-CHMICA. Forensic Sci Int.

[16] Howlett AC, Barth F, Bonner TI, Cabral G, Casellas P, Devane WA (2002). International union of pharmacology. XXVII. Classification of cannabinoid receptors. Pharmacol Rev.

[17] Pertwee RG, Howlett AC, Abood ME, Alexander SPH, Marzo VD, Elphick MR (2010). International union of basic and clinical pharmacology. LXXIX. Cannabinoid receptors and their ligands: beyond CB1 and CB2. Pharmacol Rev.

[18] Ashton JC, Friberg D, Darlington CL, Smith PF (2006). Expression of the cannabinoid CB2 receptor in the rat cerebellum: an immunohistochemical study. Neurosci Lett.

[19] Van Sickle MD, Duncan M, Kingsley PJ, Mouihate A, Urbani P, Mackie K (2005). Identification and functional characterization of brainstem cannabinoid CB2 receptors. Science.

[20] Pertwee RG (2000). Cannabinoid receptor ligands: clinical and neuropharmacological considerations, relevant to future drug discovery and development. Expert Opin Investig Drugs.

[21] Howlett AC. Cannabinoid Receptor Signaling. In: Pertwee RG, editor. Cannabinoids. Heidelberg: Springer Berlin Heidelberg; 2005. p. 53–79. Doi: 10.1007/3-540-26573-2_2

[22] Turu G, Hunyady L (2010). Signal transduction of the CB1 cannabinoid receptor. J Mol Endocrinol.

[23] Zou S, Kumar U (2018). Cannabinoid receptors and the endocannabinoid system: signaling and function in the central nervous system. Int J Mol Sci.

[24] Gallelli CA, Calcagnini S, Romano A, Koczwara JB, De Ceglia M, Dante D (2018). Modulation of the oxidative stress and lipid peroxidation by endocannabinoids and their lipid analogues. Antioxidants.

[25] Roque-Bravo R, Silva RS, Malheiro RF, Carmo H, Carvalho F, da Silva DD (2023). Synthetic cannabinoids: a pharmacological and toxicological overview. Annu Rev Pharmacol Toxicol.

[26] Morales P, Jagerovic N (2016). Advances towards the discovery of GPR55 ligands. Curr Med Chem.

[27] Guerrero-Alba R, Barragán-Iglesias P, González-Hernández A, Valdez-Moráles EE, Granados-Soto V, Condés-Lara M (2019). Some Prospective alternatives for treating pain: the endocannabinoid system and its putative receptors GPR18 and GPR55. Front Pharmacol.

[28] Di Marzo V, De Petrocellis L (2010). Endocannabinoids as regulators of transient receptor potential (TRP) channels: a further opportunity to develop new endocannabinoid-based therapeutic drugs. Curr Med Chem.

[29] O’Sullivan SE (2007). Cannabinoids go nuclear: evidence for activation of peroxisome proliferator-activated receptors. Br J Pharmacol.

[30] Lu H, Mackie K (2016). An introduction to the endogenous cannabinoid system. Biol Psychiatry.

[31] Basavarajappa BS, Subbanna S (2014). CB1 receptor-mediated signaling underlies the hippocampal synaptic, learning, and memory deficits following treatment with JWH-081, a new component of spice/K2 preparations. Hippocampus.

[32] Zawilska JB, Wojcieszak J (2014). Spice/K2 drugs–more than innocent substitutes for marijuana. Int J Neuropsychopharmacol.

[33] Cohen K, Mama Y, Rosca P, Pinhasov A, Weinstein A (2020). Chronic use of synthetic cannabinoids is associated with impairment in working memory and mental flexibility. Front Psychiatry..

[34] Spaderna M, Addy PH, D’Souza DC (2013). Spicing things up: synthetic cannabinoids. Psychopharmacology.

[35] Fattore L (2016). Synthetic cannabinoids-further evidence supporting the relationship between cannabinoids and psychosis. Biol Psychiatry Biol Psychiatry.

[36] Walsh K, Andersen H (2020). Molecular pharmacology of synthetic cannabinoids: delineating CB1R receptor-mediated cell signaling. Int J Mol Sci.

[37] Patel M, Manning JJ, Finlay DB, Javitch JA, Banister SD, Grimsey NL (2020). Signalling profiles of a structurally diverse panel of synthetic cannabinoid receptor agonists. Biochem Pharmacol.

[38] Tomiyama K, Funada M (2014). Cytotoxicity of synthetic cannabinoids on primary neuronal cells of the forebrain: the involvement of cannabinoid CB1 receptors and apoptotic cell death. Toxicol Appl Pharmacol.

[39] Canazza I, Ossato A, Vincenzi F, Gregori A, Di Rosa F, Nigro F (2017). Pharmaco-toxicological effects of the novel third-generation fluorinate synthetic cannabinoids, 5F-ADBINACA, AB-FUBINACA, and STS-135 in mice In vitro and in vivo studies. Human Psychopharmacol Clin Exp..

[40] Hebert-Chatelain E, Desprez T, Serrat R, Bellocchio L, Soria-Gomez E, Busquets-Garcia A (2016). A cannabinoid link between mitochondria and memory. Nature.

[41] Alexandre J, Malheiro R, Silva DD da, Carmo H, Carvalho F, Silva JP. The Synthetic Cannabinoids THJ-2201 and 5F-PB22 Enhance In Vitro CB1 receptor-mediated neuronal differentiation at biologically relevant concentrations. Int J Mol Sci. 2020; 21. https://api.semanticscholar.org/CorpusID:221466894 10.3390/ijms21176277 PMC7503567 32872617

[42] Oztas E, Abudayyak M, Celiksoz M, Özhan G (2019). Inflammation and oxidative stress are key mediators in AKB48-induced neurotoxicity in vitro. Toxicol In Vitro.

[43] Sezer Y, Jannuzzi AT, Huestis MA, Alpertunga B (2020). In vitro assessment of the cytotoxic, genotoxic and oxidative stress effects of the synthetic cannabinoid JWH-018 in human SH-SY5Y neuronal cells. Toxicol Res.

[44] Coccini T, De Simone U, Lonati D, Scaravaggi G, Marti M, Locatelli C (2021). MAM-2201, one of the most potent—naphthoyl indole derivative—synthetic cannabinoids, exerts toxic effects on human cell-based models of neurons and astrocytes. Neurotox Res.

[45] Ashton J (2012). Synthetic cannabinoids as drugs of abuse. Curr Drug Abuse Rev.

[46] Fantegrossi WE, Wilson CD, Berquist MD (2018). Pro-psychotic effects of synthetic cannabinoids: interactions with central dopamine, serotonin, and glutamate systems. Drug Metab Rev.

[47] Ossato A, Uccelli L, Bilel S, Canazza I, Di Domenico G, Pasquali M (2017). Psychostimulant effect of the synthetic cannabinoid JWH-018 and AKB48: behavioral, neurochemical, and dopamine transporter scan imaging studies in mice. Front Psychiatry..

[48] Gessa GL, Casu MA, Carta G, Mascia MS (1998). Cannabinoids decrease acetylcholine release in the medial-prefrontal cortex and hippocampus, reversal by SR 141716A. Eur J Pharmacol.

[49] Franklin JM, Carrasco GA (2012). Cannabinoid-induced enhanced interaction and protein levels of serotonin 5-HT2A and dopamine D2 receptors in rat prefrontal cortex. J Psychopharmacol.

[50] Franklin JM, Mathew M, Carrasco GA (2013). Cannabinoid-induced upregulation of serotonin 2A receptors in the hypothalamic paraventricular nucleus and anxiety-like behaviors in rats. Neurosci Lett.

[51] Brown TM, Brotchie JM, Fitzjohn SM (2003). Cannabinoids decrease corticostriatal synaptic transmission via an effect on glutamate uptake. J Neurosci.

[52] Liu Q, Bhat M, Bowen WD, Cheng J (2009). Signaling Pathways from cannabinoid receptor-1 activation to inhibition of <em>N</em>-Methyl-<span class="sc">d</span>-aspartic acid mediated calcium influx and neurotoxicity in dorsal root ganglion neurons. J Pharmacol Exp Ther.

[53] Lauckner JE, Jensen JB, Chen H-Y, Lu H-C, Hille B, Mackie K (2008). GPR55 is a cannabinoid receptor that increases intracellular calcium and inhibits M current. Proc Natl Acad Sci.

[54] Leo LM, Familusi B, Hoang M, Smith R, Lindenau K, Sporici KT (2019). GPR55-mediated effects on brain microvascular endothelial cells and the blood–brain barrier. Neuroscience.

[55] Saliba SW, Jauch H, Gargouri B, Keil A, Hurrle T, Volz N (2018). Anti-neuroinflammatory effects of GPR55 antagonists in LPS-activated primary microglial cells. J Neuroinflammation.

[56] Li W-J, Shen J (2022). Antagonism of G protein-coupled receptor 55 prevents lipopolysaccharide-induced damages in human dental pulp cells. Hum Exp Toxicol.

[57] Apweiler M, Saliba SW, Streyczek J, Hurrle T, Gräßle S, Bräse S (2021). Targeting oxidative stress: novel coumarin-based inverse agonists of GPR55. Int J Mol Sci.

[58] Reyes-Resina I, Navarro G, Aguinaga D, Canela EI, Schoeder CT, Załuski M (2018). Molecular and functional interaction between GPR18 and cannabinoid CB2 G-protein-coupled receptors. Relevance in neurodegenerative diseases. Biochem Pharmacol.

[59] O’Sullivan SE (2016). An update on PPAR activation by cannabinoids. Br J Pharmacol.

[60] Iannotti FA, Vitale RM (2021). The endocannabinoid system and PPARs: focus on their signalling crosstalk, action and transcriptional regulation. Cells.

[61] Andersen H, Walsh K (2021). Molecular signaling of synthetic cannabinoids: comparison of CB1 receptor and TRPV1 channel activation. Eur J Pharmacol.

[62] Wood DM, Hill SL, Thomas SHL, Dargan PI (2014). Using poisons information service data to assess the acute harms associated with novel psychoactive substances. Drug Test Anal.

[63] Atik SU, Dedeoglu R, Varol F, Çam H, Eroğlu AG, Saltık L (2015). Cardiovascular side effects related with use of synthetic cannabinoids “bonzai” : two case reports. Turk pediatri arsivi.

[64] Obafemi AI, Kleinschmidt K, Goto C, Fout D (2015). Cluster of acute toxicity from ingestion of synthetic cannabinoid-laced brownies. J Med Toxicol.

[65] Lam RPK, Tang MHY, Leung SC, Chong YK, Tsui MSH, Mak TWL (2017). Supraventricular tachycardia and acute confusion following ingestion of e-cigarette fluid containing AB-FUBINACA and ADB-FUBINACA: a case report with quantitative analysis of serum drug concentrations. Clin Toxicol.

[66] Mir A, Obafemi A, Young A, Kane C (2011). Myocardial infarction associated with use of the synthetic cannabinoid K2. Pediatrics.

[67] Ibrahim S, Al-Saffar F, Wannenburg T (2014). A Unique case of cardiac arrest following K2 abuse. Case Reports Cardiol..

[68] Davis C, Boddington D (2015). Teenage cardiac arrest following abuse of synthetic cannabis. Heart Lung Circ.

[69] McIlroy G, Ford LT, Khan JM (2016). Acute myocardial infarction, associated with the use of a synthetic adamantyl-cannabinoid: a case report. BMC Pharmacol Toxicol.

[70] Ahmed T, Khan A, See VY, Robinson SW (2020). Cardiac arrest associated with synthetic cannabinoid use and acquired prolonged QTc interval: a case report and review of literature. HeartRhythm Case Reports.

[71] Labay LM, Caruso JL, Gilson TP, Phipps RJ, Knight LD, Lemos NP (2016). Synthetic cannabinoid drug use as a cause or contributory cause of death. Forensic Sci Int.

[72] Patton AL, Chimalakonda KC, Moran CL, McCain KR, Radominska-Pandya A, James LP (2013). K2 toxicity: fatal case of psychiatric complications following AM2201 exposure. J Forensic Sci.

[73] Anzillotti L, Marezza F, Calò L, Banchini A, Cecchi R (2019). A case report positive for synthetic cannabinoids: are cardiovascular effects related to their protracted use?. Leg Med.

[74] Darke S, Duflou J, Farrell M, Peacock A, Lappin J (2020). Characteristics and circumstances of synthetic cannabinoid-related death. Clin Toxicol.

[75] Boland DM, Reidy LJ, Seither JM, Radtke JM, Lew EO (2020). Forty-three fatalities involving the synthetic cannabinoid, 5-fluoro-ADB: forensic pathology and toxicology implications. J Forensic Sci.

[76] Pacher P, Steffens S, Haskó G, Schindler TH, Kunos G (2018). Cardiovascular effects of marijuana and synthetic cannabinoids: the good, the bad, and the ugly. Nat Rev Cardiol.

[77] Radaelli D, Manfredi A, Zanon M, Fattorini P, Scopetti M, Neri M (2021). Synthetic cannabinoids and cathinones cardiotoxicity: facts and perspectives. Curr Neuropharmacol.

[78] Varga ZV, Ferdinandy P, Liaudet L, Pacher P (2015). Drug-induced mitochondrial dysfunction and cardiotoxicity. Am J Physiol Heart Circul Physiol.

[79] Alexandre J, Malheiro R, da Dias Silva D, Carmo H, Carvalho F, Silva JP (2020). The synthetic cannabinoids THJ-2201 and 5F-PB22 enhance in vitro CB1 receptor-mediated neuronal differentiation at biologically relevant concentrations. Int J Mol Sci.

[80] Silva JP, Araújo AM, de Pinho PG, Carmo H, Carvalho F (2019). Synthetic cannabinoids JWH-122 and THJ-2201 disrupt endocannabinoid-regulated mitochondrial function and activate apoptotic pathways as a primary mechanism of in vitro nephrotoxicity at in vivo relevant concentrations. Toxicol Sci.

[81] Kaminski NE (1998). Inhibition of the cAMP signaling cascade via cannabinoid receptors: a putative mechanism of immune modulation by cannabinoid compounds. Toxicol Lett.

[82] Rahman N, Buck J, Levin L (2013). pH sensing via bicarbonate-regulated “soluble” adenylyl cyclase (sAC). Front Physiol.

[83] Singh A, Saluja S, Kumar A, Agrawal S, Thind M, Nanda S (2018). Cardiovascular complications of marijuana and related substances: a review. Cardiol Therapy.

[84] Sierra S, Luquin N, Navarro-Otano J (2018). The endocannabinoid system in cardiovascular function: novel insights and clinical implications. Clin Auton Res.

[85] Puhl S-L, Hilby M, Kohlhaas M, Keidel LM, Jansen Y, Hristov M (2021). Haematopoietic and cardiac GPR55 synchronize post-myocardial infarction remodelling. Sci Rep.

[86] Park F, Potukuchi PK, Moradi H, Kovesdy CP (2017). Cannabinoids and the kidney: effects in health and disease. Am J Physiol Renal Physiol.

[87] Murphy TD, Weidenbach KN, Van Houten C, Gerona RR, Moran JH, Kirschner RI, Marraffa JM, Stork CM, Birkhead GS, Newman A, Hendrickson R (2013). Centers for Disease Control and Prevention (CDC). Acute kidney injury associated with synthetic cannabinoid use—multiple states, 2012. MMWR Morb Mortal Wkly Rep.

[88] Bhanushali GK, Jain G, Fatima H, Leisch LJ, Thornley-Brown D. AKI Associated with Synthetic Cannabinoids: A Case Series. Clinical Journal of the American Society of Nephrology. 2013. https://journals.lww.com/cjasn/Fulltext/2013/04000/AKI_Associated_with_Synthetic_Cannabinoids__A_Case.5.aspx 10.2215/CJN.05690612 PMC3613952 23243266

[89] Kazory A, Aiyer R (2013). Synthetic marijuana and acute kidney injury: an unforeseen association. Clin Kidney J.

[90] Gudsoorkar VS, Perez Jose A (2015). A New differential diagnosis: synthetic cannabinoids-associated acute renal failure. Methodist DeBakey Cardiovas J..

[91] D’Errico S, Zanon M, Radaelli D, Concato M, Padovano M, Scopetti M (2022). Acute kidney injury (AKI) in young synthetic cannabinoids abusers. Biomedicines..

[92] Lin C-L, Hsu Y-C, Lee P-H, Lei C-C, Wang J-Y, Huang Y-T (2014). Cannabinoid receptor 1 disturbance of PPARγ2 augments hyperglycemia induction of mesangial inflammation and fibrosis in renal glomeruli. J Mol Med.

[93] Nam DH, Lee MH, Kim JE, Song HK, Kang YS, Lee JE (2012). Blockade of cannabinoid receptor 1 improves insulin resistance, lipid metabolism, and diabetic nephropathy in db/db mice. Endocrinology.

[94] Silva GB, Atchison DK, Juncos LI, García NH (2013). Anandamide inhibits transport-related oxygen consumption in the loop of Henle by activating CB1 receptors. Am J Physiol Renal Physiol.

[95] Ritter JK, Li G, Xia M, Boini KM (2016). Anandamide and its metabolites: what are their roles in the kidney?. Front Biosci.

[96] Tam J (2016). The emerging role of the endocannabinoid system in the pathogenesis and treatment of kidney diseases. J Am Soc Nephrol.

[97] Silva JP, Carmo H, Carvalho F (2018). The synthetic cannabinoid XLR-11 induces in vitro nephrotoxicity by impairment of endocannabinoid-mediated regulation of mitochondrial function homeostasis and triggering of apoptosis. Toxicol Lett.

[98] Lim JC, Lim SK, Han HJ, Park SH (2010). Cannabinoid receptor 1 mediates palmitic acid-induced apoptosis via endoplasmic reticulum stress in human renal proximal tubular cells. J Cell Physiol.

[99] Mukhopadhyay P, Pan H, Rajesh M, Bátkai S, Patel V, Harvey-White J (2010). CB1 cannabinoid receptors promote oxidative/nitrosative stress, inflammation and cell death in a murine nephropathy model. Br J Pharmacol.

[100] Takayama T, Suzuki M, Todoroki K, Inoue K, Min JZ, Kikura-Hanajiri R (2014). UPLC/ESI-MS/MS-based determination of metabolism of several new illicit drugs, ADB-FUBINACA, AB-FUBINACA, AB-PINACA, QUPIC, 5F-QUPIC and α-PVT, by human liver microsome. Biomed Chromatogr.

[101] Hsin-Hung Chen M, Dip A, Ahmed M, Tan ML, Walterscheid JP, Sun H (2016). Detection and characterization of the effect of AB-FUBINACA and its metabolites in a rat model. J Cell Biochem.

[102] Alzu’bi A, Zoubi MS, Al-Trad B, AbuAlArjah MI, Shehab M, Alzoubi H (2022). Acute hepatic injury associated with acute administration of synthetic cannabinoid XLR-11 in mouse animal model. Toxics..

[103] Sheikh IA, Lukšič M, Ferstenberg R, Culpepper-Morgan JA (2014). SPICE/K2 synthetic marijuana-induced toxic hepatitis treated with N-acetylcysteine. Am J Case Rep.

[104] Paez M, Laiyemo A, Atanda AC, Mehari A, Davis W, Odeyemi Y. Synthetic Marijuana-Induced Acute Liver Failure: 1820. Official journal of the American College of Gastroenterology | ACG. 2016. https://journals.lww.com/ajg/Fulltext/2016/10001/Synthetic_Marijuana_Induced_Acute_Liver_Failure_.1820.aspx

[105] Etienne D, Ofori E, Mullangi S, Shah J, Ona MA, Stevens M, et al. A Case of Synthetic Marijuana (Spice/K2)-induced Liver Injury: 1772. Official journal of the American College of Gastroenterology | ACG. 2016. https://journals.lww.com/ajg/Fulltext/2016/10001/A_Case_of_Synthetic_Marijuana__Spice_K2__induced.1772.aspx

[106] Knowles KJ, Wei EX, Seth A, Bienvenu J, Morris J, Manas K, et al. Synthetic Cannabinoid Abuse and a Rare Alpha-1-Antitrypsin Mutant Causing Acute Fulminant Hepatitis: A Case Report and Review of the Literature. Case Reports Hepatol. 2017. https://link.gale.com/apps/doc/A550492649/HRCA?u=anon~13c915bd&sid=googleScholar&xid=bf33902b. Accessed 19 May 2023 10.1155/2017/9627452 PMC5733121 29333304

[107] Behonick G, Shanks KG, Firchau DJ, Mathur G, Lynch CF, Nashelsky M (2014). Four postmortem case reports with quantitative detection of the synthetic cannabinoid, 5F-PB-22. J Anal Toxicol.

[108] Gonzalez-Gonzalez FJ, Chandel NS, Jain M, Budinger GRS (2017). Reactive oxygen species as signaling molecules in the development of lung fibrosis. Transl Res.

[109] Liu J, Gao B, Mirshahi F, Sanyal AJ, Khanolkar AD, Makriyannis A (2000). Functional CB1 cannabinoid receptors in human vascular endothelial cells. Biochem J.

[110] Parfieniuk A, Flisiak R (2008). Role of cannabinoids in chronic liver diseases. World J Gastroenterol.

[111] Kim Y, Gautam S, Aseer KR, Kim J, Chandrasekaran P, Mazucanti CH (2020). Hepatocyte cannabinoid 1 receptor nullification alleviates toxin-induced liver damage via NF-κB signaling. Cell Death Dis.

[112] Azar S, Udi S, Drori A, Hadar R, Nemirovski A, Vemuri KV (2020). Reversal of diet-induced hepatic steatosis by peripheral CB1 receptor blockade in mice is p53/miRNA-22/SIRT1/PPARα dependent. Mol Metabol.

[113] Alon MH, Saint-Fleur MO (2017). Synthetic cannabinoid induced acute respiratory depression: case series and literature review. Respir Med Case Rep.

[114] Nagre N, Nicholson G, Cong X, Pearson A, Hattler J, Kim W-K, et al. Activation of Cannabinoid-2 Receptor Protects Against Pseudomonas Aeruginosa Induced Acute Lung Injury and Inflammation. TP113 TP113 Acute lung injury and repair. American Thoracic Society; 2021. p. A4363–A4363. 10.1164/ajrccm-conference.2021.203.1_MeetingAbstracts.A4363.Accessed 5 Oct 2023.

[115] Śledziński P, Nowak-Terpiłowska A, Zeyland J (2021). Cannabinoids in medicine: cancer, immunity, and microbial diseases. Int J Mol Sci.

[116] Wang MTM, Danesh-Meyer HV (2021). Cannabinoids and the eye. Surv Ophthalmol.

[117] Ortiz-Peregrina S, Ortiz C, Casares-López M, Jiménez JR, Anera RG (2021). Effects of cannabis on visual function and self-perceived visual quality. Sci Rep.

